# Waste Biomass-Mediated Synthesis of TiO_2_/P, K-Containing Grapefruit Peel Biochar Composites with Enhanced Photocatalytic Activity

**DOI:** 10.3390/molecules29092090

**Published:** 2024-05-01

**Authors:** Ruixiang Wu, Wenhua Liu, Renao Bai, Delun Zheng, Xiufang Tian, Weikai Lin, Qianwei Ke, Lejian Li

**Affiliations:** 1 Guangdong Provincial Laboratory of Marine Biotechnology, Institute of Marine Sciences, Shantou University, Shantou 515063, China; rxwu1@stu.edu.cn; 2 College of Construction and Ecology, Shantou Polytechnic, Shantou 515078, China; rabai@stpt.edu.cn (R.B.); xftian@stpt.edu.cn (X.T.); linweikai@163.com (W.L.); qianweike1999@163.com (Q.K.); lilejian2024@163.com (L.L.)

**Keywords:** biomimetic synthesis, rotary impregnation, TiO_2_/P, K-PC, photocatalyst

## Abstract

In this study, TiO_2_/P, K-containing grapefruit peel biochar (TiO_2_/P, K-PC) composites were synthesized *in situ* biomimetically using grapefruit peel as the bio-template and carbon source and tetrabutyl titanate as the titanium source. This was achieved using the two-step rotary impregnation–calcination method. Adjusting the calcination temperature of the sample in an air atmosphere could regulate the mass ratio of TiO_2_ to carbon. The prepared samples were subjected to an analysis of their compositions, structures, morphologies, and properties. It demonstrated that the prepared samples were complexes of anatase TiO_2_ and P, K-containing carbon, with the presence of graphitic carbon. They possessed a unique morphological structure with abundant pores and a large surface area. The grapefruit peel powder played a crucial role in the induction and assembly of TiO_2_/P, K-PC composites. The sample PCT-400-550 had the best photocatalytic activity, with the degradation rate of RhB, MO, and MB dye solutions reaching more than 99% within 30 min, with satisfactory cyclic stability. The outstanding photocatalytic activity can be credited to its unique morphology and the efficient collaboration between TiO_2_ and P, K-containing biochar.

## 1. Introduction

Water is the core of sustainable development. It is crucial for human survival, energy and food security, and economic and social development. However, the rapid growth of global industry, modern agriculture, and urbanization has led to increasingly severe water pollution, posing significant limitations on sustainable social development [1]. Dye wastewater is a harmful wastewater generated during industrial manufacturing processes [2]. It is characterized by large discharge, complex composition, high chromaticity, high toxicity, and low degradability [3]. The accumulation of dyestuffs in organisms has potential hazards such as carcinogenicity, teratogenicity, and mutagenicity [4]. Currently, adsorption [5], extraction [6], micro-biological degradation [7], and photocatalysis [8] are being used for treating dye wastewater. Among these, the photocatalytic technique is favored by researchers owing to its economic advantages, environmental protection, and sustainability [9].

Photocatalytic technology fundamentally depends on the development of efficient and inexpensive photocatalysts. Design and development of affordable and environmentally friendly photocatalytic materials with high catalytic activity is crucial for the protection of the health and safety of the water environment [10]. Titanium dioxide (TiO_2_) is a widely studied photocatalyst owing to its advantages of high photocatalytic activity, good chemical stability, safety, and non-toxicity [11]. It is widely used in the degradation of organic pollutants [12], water splitting [13], CO_2_ reduction [14], and sterilization [15]. However, the application of TiO_2_ still has some bottlenecks that are primarily characterized by its wider band gap [16], higher electron–hole recombination rate [17], difficult charge separation and recovery, and easy cause of secondary pollution [18]. Researchers have investigated various methods to solve the problems mentioned above, and the preparation of TiO_2_-based composites is a commonly used method.

Biomass has the advantages of abundance, affordability, and renewability. Bio-carbon is prepared from biomass. It possesses a layered porous arrangement, a large specific surface area, and plentiful pores [19], which makes it an ideal adsorption material in wastewater treatment [20]. TiO_2_-biochar composites have many attractive properties and advantages. Recently, the preparation and application of TiO_2_-biochar composites have been widely studied. Zhang et al. [21] prepared C/TiO_2_ composites by depositing TiO_2_ on grapefruit peel biochar. These composites catalyze tetracycline degradation under simulated sunlight with excellent performance. Thuan et al. [22] prepared a TiO_2_/rice husk biochar (TiO_2_/BC) composite that achieves a 97.6% removal rate for bisphenol A within 1 h under ultraviolet (UV) light. Xiong et al. [23] prepared a N-TiO_2_/N-biochar composite using walnut husk biochar. Under mercury lamp irradiation, the photocatalytic degradation rate of methyl orange reached 97.6% within 100 min, with a mineralization rate of 85.4%. Grapefruit peel biochar has a rich pore structure and contains various organic functional groups on its surface [24]. Thus, it has potential application in the field of environmental treatment [25]. However, reports on preparing TiO_2_/biochar composites with photocatalytic activity using grapefruit peel as a bio-template and carbon source are scarce. Consequently, the preparation method of TiO_2_/biochar composites from grapefruit peels and their performance should be fully investigated.

In this study, a rotary impregnation–calcination approach was utilized to fabricate the TiO_2_/P, K-BC composites using bio-waste grapefruit peel as both the bio-template and carbon source and tetrabutyl titanate as precursor. The decision to utilize grapefruit peel as both the bio-template and carbon source stems from findings in our prior research, where it was transformed into porous biochar rich in abundant phosphorus and potassium elements, thereby promoting the nucleation, growth, and deposition of TiO_2_ nanoparticles. We adjusted the calcination temperature of the samples in the air to tune the content of biochar in the composites and prepared a series of TiO_2_/P, K-PC composites. This paper also investigated the photocatalytic activities in the degradation of dye wastewater and delved into the synthesis mechanism of the TiO_2_/P, K-PC composites. Our work elucidated the *in situ* nucleation process and growth of TiO_2_ on grapefruit peel. This approach not only made it possible to regulate and control the morphology and structure of the TiO_2_/P, K-PC composites, but it also provided a source of P and K-containing biochar from the grapefruit peel. This method represents a sustainable, cost-effective, and controllable strategy for achieving green and efficient synthesis.

## 2. Results and Discussion

### 2.1. Composition Analysis of Prepared Samples

**Figure 1:**
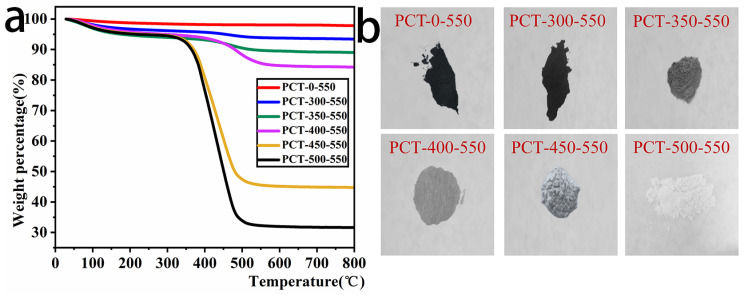
TGA curves (**a**) and physical images (**b**) of TiO_2_/P, K-PC composites.

To investigate the relative contents of TiO_2_ and biochar in the composites, thermogravimetric analysis (TGA) was performed on the prepared samples. Figure 1 depicts the obtained TGA curves of the prepared samples in an air atmosphere at temperatures starting from room temperature up to 800 °C at a heating rate of 5 °C/min and the physical image of the samples. As shown in Figure 1a, the thermogravimetric behavior of the prepared samples mainly underwent two stages. The first stage was from room temperature to 150 °C, which is due to the moisture adsorbed on the surface, and the second stage was from 300 °C to nearly 500 °C, which is due to the biochar combustion. Based on the TGA results, it can be deduced that the contents of carbon in the PCT-x-550 samples were as follows: PCT-0-550: 64.14%, PCT-300-550: 51.15%, PCT-350-550: 12.18%, PCT-400-550: 6.39%, PCT-450-550: 3.76%, and PCT-500-550 (the content of carbon can be ignored).

The color change in PCT-x-550 samples was dark black → black → dark grey → light grey → off-white → white (Figure 1b). From these TGA analysis results and the observed color change in the samples, it can be seen that the content of bio-carbon in the PCT-x-550 samples decreases with the increase in their corresponding calcination temperatures in the air atmosphere. Therefore, the carbon content in the composites can be regulated by adjusting the calcination temperature of the sample in air.

### 2.2. Structural Analysis of Prepared Samples

**Figure 2:**
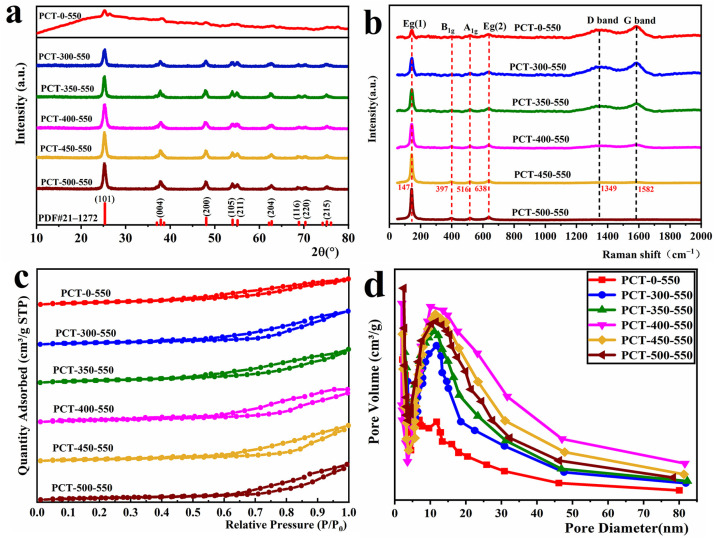
XRD spectra (**a**), Raman spectra (**b**), adsorption–desorption curve of N_2_ (**c**), and pore size distribution (**d**) of TiO_2_/P, K-PC composites.

From the X-ray diffraction (XRD) spectrum of TiO_2_/P, K-PC composites (Figure 2a), it can be seen that the diffraction peaks of PCT-x-550 (x = 0, 300, 350, 400, 450, 500) samples at 2θ = 25.26°, 37.82°, 48.03°, 53.91°, 55.03°, 62.68°, 68.83°, 70.25°, and 75.14° correspond to the (101), (004), (200), (105), (211), (204), (116), (220), and (215) crystal faces in the standard diffraction of anatase TiO_2_ (PDF#21-1272) [26]. Moreover, the intensity of each diffraction peak enhanced gradually as the x-value increased. This is because the TiO_2_ content in the composites gradually increases as the x-value increases. Additionally, the PCT-0-550 sample showed a broad peak between 2θ = 20.00° and ~30.00° and a narrow peak at 26.34°, which are characteristic peaks of amorphous and graphitized carbon, respectively (JCPDS41-1487) [27]. This indicates that grapefruit peel biomass was converted to biochar by calcination under the N_2_ atmosphere. Using Scherrer’s formula [28], the particle sizes of TiO_2_ in the PCT-x-550 (x = 0, 300, 350, 400, 450, 500) samples were calculated to be 12.63 nm, 15.12 nm, 16.83 nm, 17.52 nm, 18.32 nm, and 21.28 nm, respectively. With increasing x-value, the particle size of TiO_2_ gradually increases, which reveals that the higher biochar content in the samples inhibits the growth of TiO_2_ grains. Thus, the results of the analysis of XRD spectroscopy prove that TiO_2_/PC composites were successfully synthesized.

To further investigate the carbon and overall structural information in the TiO_2_/P, K-PC composites, Raman spectrum testing was performed on the PCT-x-550 (x = 0, 300, 350, 400, 450, 500) samples. The obtained results are shown in Figure 2b. As shown, four peaks were observed at 147 cm^−1^, 397 cm^−1^, 516 cm^−1^, and 638 cm^−1^, which correspond to the Raman peaks of E g (1), B1g, A1g, and E g (2) of anatase TiO_2_, respectively [29], indicating the formation of anatase TiO_2_ in the PCT-x-550 samples. The Raman peaks of TiO_2_ gradually enhanced as the x-value increased, implying that the TiO_2_ content in PCT-x-550 samples gradually increased. Additionally, two typical Raman peaks were observed at 1349 cm^−1^ and 1582 cm^−1^. The former refers to the D band associated with the structural defects of carbon and the induction of graphite disorder, and the latter is the G band corresponding to the intrinsic Raman peak of graphitic carbon. Furthermore, the D and G bands occur due to the sp^3^ and sp^2^ hybridization of carbon atoms, respectively [30,31]. The intensities of the D and G peaks decrease gradually as the x-value increases, indicating that the graphitic carbon content in PCT-x-550 (x = 0, 300, 350, 400, 450, 500) samples gradually decreases as their calcination temperatures increase in the air atmosphere. Moreover, because graphitic carbon has excellent electron transport ability, its presence in TiO_2_/P, K-PC composites is beneficial for mitigating the recombination rate of electron–hole pairs and enhancing their photocatalytic efficiency.

The results of the Raman spectrum analysis showed the presence of Raman peaks of anatase structure TiO_2_ and carbon in PCT-x-550 samples. Combining these results with the XRD analysis results proves that the PCT-x-550 samples were TiO_2_-biochar composites, and the carbon in the sample PCT-500-550 was negligible.

In order to analyze the pore structure and surface area of the PCT-x-550 (x = 0, 300, 350, 400, 450, 500) samples, an adsorption–desorption test of nitrogen was conducted on them. The obtained results are depicted in Figure 2c. According to the classification rules of IUPAC [32], the nitrogen adsorption–desorption isotherms of all of these samples (Figure 2c) were of type IV. They exhibited the H3-type hysteresis loop, indicating that all the PCT-x-550 samples had typical mesoporous structures. From the pore size distribution diagram of the samples (Figure 2d), it can be seen that the pore size of PCT-x-550 samples is primarily concentrated in the 3 nm-30 nm range, which is a typical mesoporous pore size. The data of surface area, pore volume, and average pore size of TiO_2_/P, K-PC composites are provided in Table 1. The surface area, pore volume, and average pore size of the samples showed a trend of initial increase followed by a decline as the x-value increased. The sample PCT-400-550 had the largest surface area, pore volume, and average pore size. Thus, it has the potential advantage of becoming an excellent photocatalyst.

### 2.3. Surface Elemental Analysis of Prepared Samples

**Figure 3:**
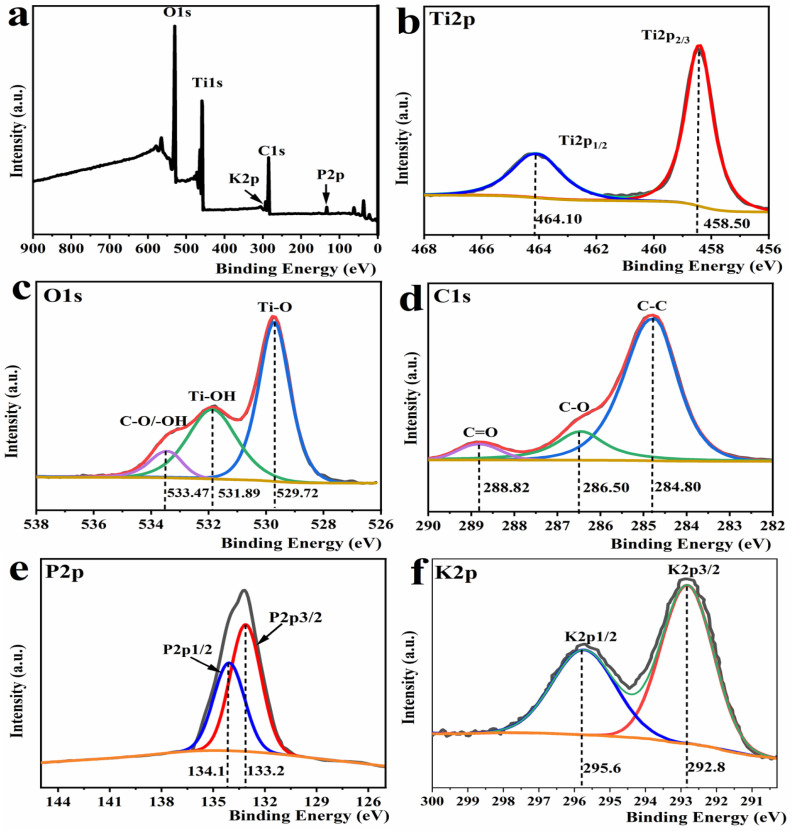
XPS full spectrum (**a**), high-resolution XPS spectrum of Ti 2p (**b**), O 1s (**c**), C 1s (**d**), P2p (**e**), and K2p (**f**) of the sample PCT-400-550.

To examine the elemental composition on the surface of the samples, as well as their chemical valence states, the sample PCT-400-550 was subjected to X-ray spectroscopy (XPS) analysis. The obtained results are depicted in Figure 3.

The XPS full spectrum image in Figure 3a shows that PCT-400-550 contains five elements: Ti, O, C, P, and K. The high-resolution spectrum of Ti 2p (Figure 3b) was fitted using Gaussian splitting to two peaks at 458.50 eV and 464.10 eV, which are the characteristic peaks of Ti 2p_1/2_ and Ti 2p_3/2_, respectively [33]. This indicates that the Ti element in the PCT-400-550 samples exists in the form of Ti^4+^ [34]. Combining these results with the XRD spectrum shown in Figure 2a, it was observed that TiO_2_ crystals in anatase were generated in the PCT-400-550 samples [35]. The high-resolution spectrum of O 1s shown in Figure 3c was deconvoluted to three peaks at 533.47 eV, 531.89 eV, and 529.72 eV, which belong to the oxygen elements in the C-O, Ti-OH, and Ti-O bonds, respectively [36]. The strong peaks at 529.72 eV and 531.89 eV correspond to Ti-O and Ti-OH peaks, respectively. These may originate from -OH adsorbed on the PCT-400-550 sample surface or from Ti(O-C_4_H_9_)_4-n_(OH)_n_ generated by hydrolysis of tetrabutyl titanate, and the peak at 529.72 eV corresponds to the formation of C-O or -OH on TiO_2_ surface owing to C-O and H_2_O or -OH adsorbed on the sample surface [37]. The high resolution of C 1s shown in Figure 3d can be fitted to three peaks at 288.82 eV, 286.50 eV, and 284.80 eV, which correspond to C=O, C-O, graphitic carbon, and carbon in C-C, respectively [38]. This indicates the presence of graphitic carbon in the PCT-400-550 sample, which is consistent with the results of the Raman spectrum analysis (Figure 2b). C-O and Ti-O bonds were present in the XPS spectrum of C 1s and O 1s, respectively, and the atomic ratio of oxygen and titanium elements in the PCT-400-550 sample was approximately 3:1, which is much higher than the theoretical value of 2:1 for TiO_2_. This implied that the TiO_2_ in the PCT-400-550 sample was bonded to the carbon via a C-O-Ti bond. The high-resolution XPS spectrum of P2p (Figure 3e) can be peak-fitted into two peaks at 133.20 eV and 134.12 eV, corresponding to the P 2p3 and 2p1 orbitals, respectively, providing evidence for the existence of P-O bonds [39]. The high-resolution XPS spectrum of K2p (Figure 3f) can be peak-fitted into two peaks at 292.82 eV and 295.59 eV, corresponding to the K 2p3 and 2p1 orbitals, respectively, confirming the presence of potassium ions [40].

### 2.4. Morphological Analysis of Prepared Samples

**Figure 4:**
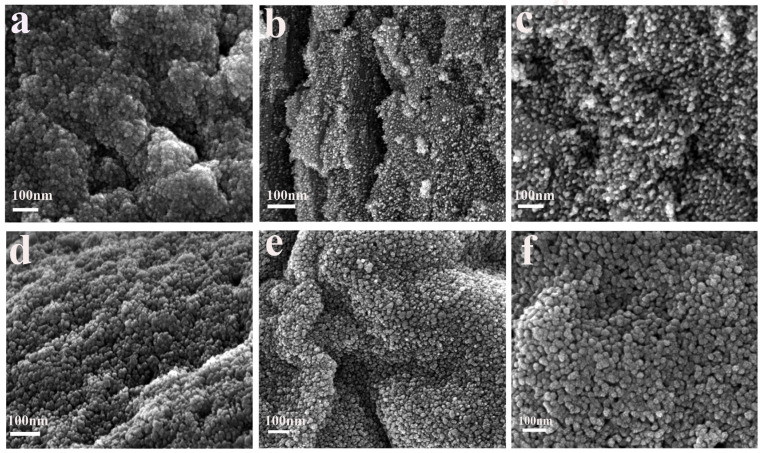
SEM images of PCT-x-550 samples: (**a**) PCT-0-550, (**b**) PCT-300-550, (**c**) PCT-350-550, (**d**) PCT-400-550, (**e**) PCT-450-550, and (**f**) PCT-500-550.

To investigate the effect of the calcination temperatures of samples in an air atmosphere on their morphology and structure, scanning electron microscope (SEM) analysis was performed on PCT-x-550 (x = 0, 300, 350, 400, 450, 500) samples. The obtained results are depicted in Figure 4.

Figure 4a shows that the TiO_2_ nanoparticles of sample PCT-0-550 were encapsulated in biochar, which caused TiO_2_ to agglomerate in a certain area, and the TiO_2_ nanoparticles blocked the biochar pores. Consequently, the surface area, the pore volume, and the average pore size of sample PCT-0-550 were relatively smaller (Table 1). Figure 4b–f show that as the carbon content in the composites decreased, the TiO_2_ nanoparticles grew outwards, from being wrapped by biochar to growing on the biochar surface and assembled according to the organizational structure of the grapefruit peel fibers. This agglomeration phenomenon gradually weakened, and the particle size gradually increased. This finding agrees with the XRD results obtained using Scherrer’s formula. When the biochar in the composites is more appropriate, its pores and those formed by the assembly of TiO_2_ nanoparticles in the sample PCT-x-550 (x = 350, 400, 450) are better presented. The surface area, pore volume, and pore size of the sample are large. This was also confirmed by its N_2_ adsorption–desorption curve (Figure 2c). When the biochar in the sample was removed, the advantage of biochar could not be shown. Consequently, the surface area, pore volume, and pore size of the sample PCT-500-550 were slightly decreased again.

**Figure 5:**
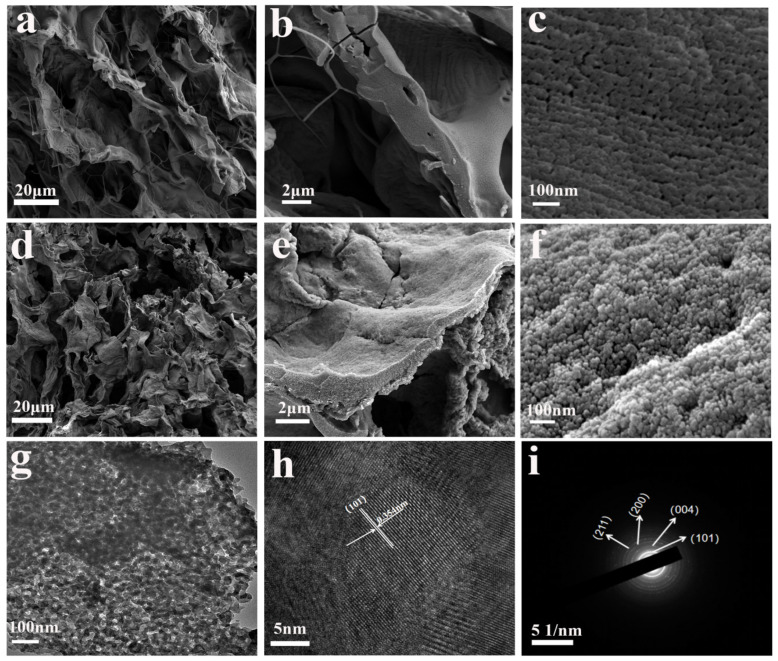
SEM images of grapefruit peel powder (**a**–**c**); SEM images of PCT-400-550 sample (**d**–**f**); TEM images (**g**), HRTEM images (**h**), and electron selected area diffraction (**i**) of PCT-400-550 sample.

To further investigate the role played by the grapefruit peel template in the formation of composites, SEM tests were conducted on grapefruit peel powder and sample PCT-400-550. The results are depicted in Figure 5.

Figure 5a–c depict SEM images of grapefruit peel powder at different magnifications. The images in Figure 5a,b show that the grapefruit peel powder has a wrinkled and porous structure with a large number of big pores in the range of 10 μm to 50 μm. The SEM image at a magnification of 10 W × (Figure 5c) reveals the presence of abundant nanoscale pores on the surface of pomelo peel fibers. And the rich pore structure makes it a potential advantage as a biological template to prepare porous material. Figure 5d–f depict that the sample PCT-400-550 perfectly replicates the morphological structure of grapefruit peel, exhibiting a surface with wrinkles and pores. From the SEM image at a magnification of 10 W× (Figure 5f), it can be observed that the sample is assembled from spheroid-shaped nanoparticles along the grapefruit peel fiber. The particle diameter is approximately 20 nm, with good dispersion. Additionally, abundant pores exist between the particles, resulting in a larger specific surface area and more active sites. This enables the materials to bind to the pollutants quickly and efficiently, thus improving their adsorption and photocatalytic efficiency.

The morphology and structure of sample PCT-400-550 were further analyzed using a transmission electron microscope (TEM). The results are depicted in Figure 5g–i. Figure 5g shows that sample PCT-400-550 consists of TiO_2_ nanoparticles and a tightly bound thin carbon layer. It is hypothesized that a heterojunction was formed at the interface between the TiO_2_ nanoparticles and biochar. The formation of this heterojunction can promote electron migration, reducing the electron–hole recombination rate in the composite, thus ultimately improving photocatalytic activity. The particle size of the nanoparticles was found to be approximately 20 nm, which is consistent with the SEM (Figure 5f) test results. In the HRTEM image (Figure 5h) of the sample PCT-400-550, lattice stripes are clearly visible, and the lattice spacing was measured to be 0.354 nm, corresponding to the (101) crystal face of anatase TiO_2_. This finding aligns with the XRD analysis outcomes. The electron-selected diffractogram image (Figure 5i) shows that the sample consists of typical polycrystalline diffraction rings, indicating that the TiO_2_ nanoparticles in the fabricated composites are polycrystalline structures with good crystallinity.

**Figure 6:**
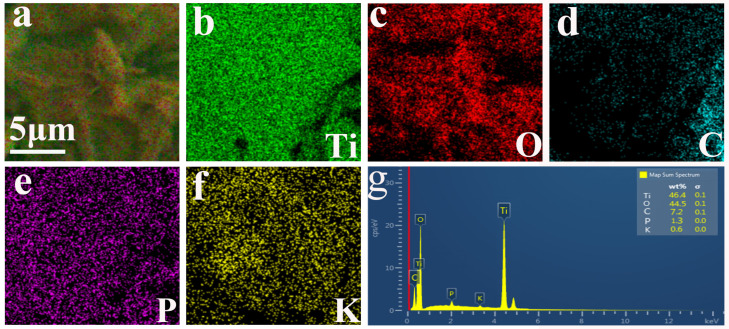
EDS elemental distributions (**a**–**f**) and EDS energy spectrum (**g**) of PCT-400-550.

Figure 6a–f depict the elemental distribution of PCT-400-550. It was observed that the Ti, O, C, P, and K elements in the PCT-400-550 sample are uniformly distributed in the whole area. The EDS energy spectrum of PCT-400-550 (Figure 6g) reveals that the weight percentage contents of Ti, O, C, P, and K in this sample are 46.4%, 44.5%,7.2%,1.3%, and 0.6%, respectively. The combined results of TGA curves (Figure 1a), XRD spectrum (Figure 2a), Raman spectra (Figure 2b), XPS (Figure 3), SEM (Figure 5), and EDS analyses (Figure 6) of the samples demonstrate well that TiO_2_/P, K-containing grapefruit peel biochar (TiO_2_/P, K-PC) composites were successfully prepared using the strategy mentioned above.

### 2.5. The Formation Mechanism of TiO_2_/P, K-PC Composites

**Figure 7:**
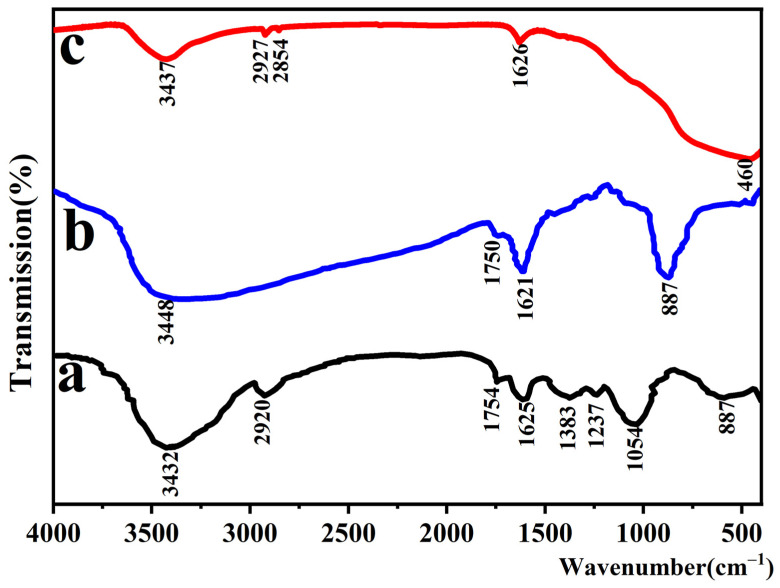
Infrared spectra of grapefruit peel powder (a), grapefruit peel powder impregnated with precursor solution (b), and sample PCT-400-550 (c).

To investigate the changes in the surface functional groups of grapefruit peel powder during the reaction process and to reveal the formation mechanism of TiO_2_/P,K-PC composites, infrared spectroscopic tests were performed on grapefruit peel powder, grapefruit peel powder impregnated with precursor liquid, and sample PCT−400−550. The obtained results are depicted in Figure 7.

In the infrared spectra of grapefruit peel powder (Figure 7a), the strong and broad characteristic peak at 3432 cm^−1^ and the absorption peak at 2920 cm^−1^ correspond to the O-H and C-H telescopic vibrations, respectively. The absorption peaks at 1754 cm^−1^ and 1625 cm^−1^ correspond to the C=O stretching vibration. The peaks at 1383 cm^−1^ and 1054 cm^−1^ are caused by the stretching vibrations of C-C and C-O-C bonds [41]. The infrared spectra of grapefruit peel powder show that the grapefruit peel surface contains abundant oxygen-containing groups, which help the grapefruit peel anchor the titanium source during the impregnation process. The infrared spectra of grapefruit peel powder impregnated with precursor solution (Figure 7b) showed that the hydroxyl absorption peak at 3448 cm^−1^ has undergone a red-shift relative to the grapefruit peel, and the absorption peak at 2920 cm^−1^ has disappeared. This is attributed to chemical reactions between Ti^4+^ in TBOT and hydroxyl, carbonyl, and other organic functional groups on the surface of grapefruit peel during the impregnation process, resulting in changes in the functional groups on the surface of the grapefruit peel. Furthermore, the FTIR spectra of sample PCT-400-550 (Figure 7c) show a distinctive absorption peak near 470 cm^−1^, which, as reported in the literature, is attributed to the stretching vibration of the Ti-O bond and the bridging stretching vibration of the Ti-O-Ti bond [42]. Additionally, the absorption peaks around 1628 cm^−1^ and 3422 cm^−1^ correspond to the stretching and bending vibrations of hydroxyl groups, which may be due to the adsorption of water or residual hydroxyl groups on the sample surface [43]. It indicates the successful preparation of TiO_2_.

**Scheme 1:**
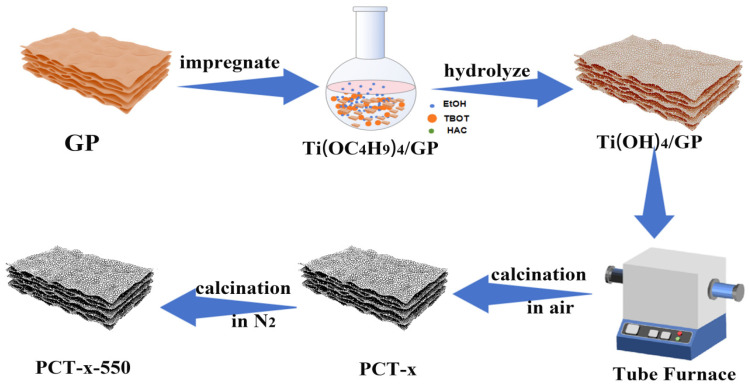
Formation process of TiO_2_/P, K-PC composites.

Based on the characterization results, sample analysis, and discussion on the analysis results, the approximate formation process of TiO_2_/P, K-PC composites is considered to be as depicted in Scheme 1. First, grapefruit peel powder was added to the alcoholic solution of tetrabutyl titanate (TBOT). Then, a prolonged rotary immersion was performed to promote the entry of the precursor liquid into the pore channels of the grapefruit peel powder, which improved the dispersion of the precursor liquid on the grapefruit peel powder. This anchored the Ti^4+^ ions to the grapefruit peel through chemical bonding or electrostatic gravitational interaction between Ti^4+^ and the functional groups, including -OH and C=O, on grapefruit peels. Grapefruit peel powder guided the precursor liquid to assemble along the grapefruit peel fibers. Consequently, Ti(OC_4_H_9_)_4_/grapefruit peel powder Ti(OC_4_H_9_)_4_/GP was generated. It was slowly hydrolyzed in contact with moisture in the air to obtain the intermediate product Ti(OH)_4_/grapefruit peel powder Ti(OH)_4_/GP *in situ* on grapefruit peel powder. Ti(OH)_4_/GP was then dried and placed in a tube furnace to be calcined under an air atmosphere. As a result, Ti(OH)_4_ decomposed on grapefruit peel powder to generate TiO_2_ *in situ*. With the increase in temperature, part of the organic matter of grapefruit peel powder generated CO_2_ and other gases that were removed, and the remainder of it was converted into bio-carbon, which was retained. Subsequently, N_2_ was introduced into the tube furnace, and the calcination of PCT-x was continued at 550 °C for 3 h under a nitrogen atmosphere to improve the crystallization degree of TiO_2_ and the graphitization degree of carbon in the composites, thereby increasing the electrical conductivity of the materials. Thus, TiO_2_/P, K-PC composites were successfully prepared.

### 2.6. Photovoltaic Performance Analysis of Prepared Samples

**Figure 8:**
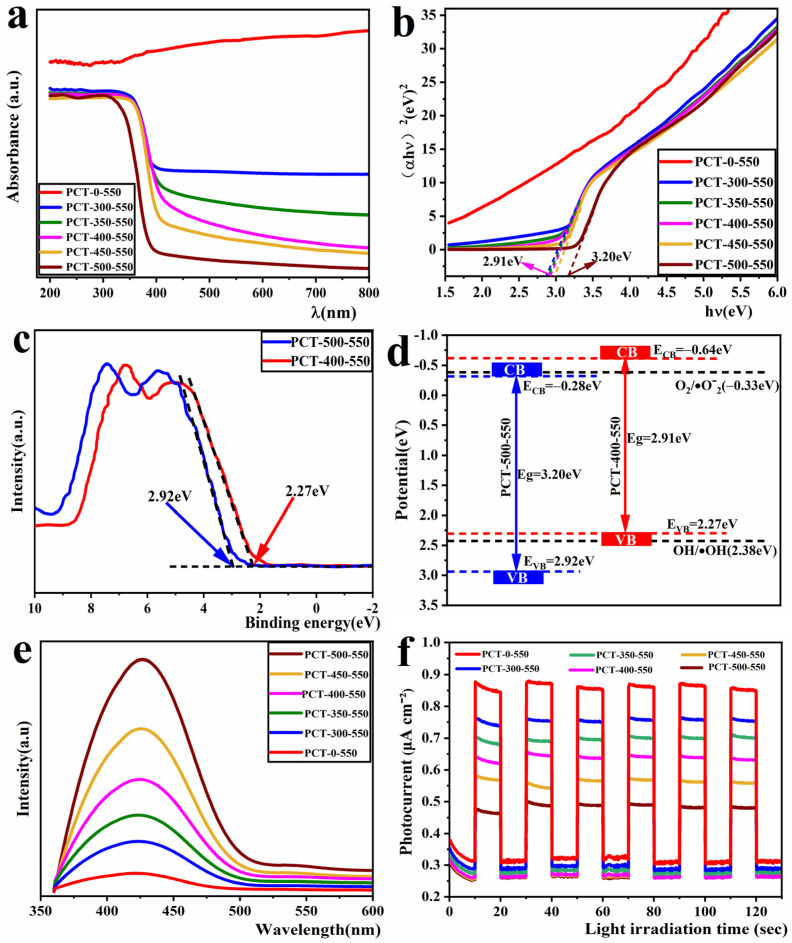
UV-Vis diffuse reflection absorption spectrum (**a**), band gap energy spectra (**b**), valence-band XPS spectra (**c**), band structures (**d**), fluorescence spectrum (**e**), and transient photocurrent response (**f**) of TiO_2_/P, K-PC composites.

The efficient light absorption ability is an important prerequisite to guarantee an excellent photocatalytic performance of the material. To investigate the light absorption ability of the samples, an ultraviolet–visible (UV-Vis) diffuse reflectance test was conducted on the prepared samples. The obtained results are depicted in Figure 8a. As shown, the sample PCT-500-550 has strong light absorption in the UV wavelength range of 200–400 nm and very weak light absorption in the visible wavelength range of 400–800 nm. This is because the main component of sample PCT-500-550 is TiO_2_ without the presence of carbon, which exhibits the light absorption characteristics of TiO_2_. The UV-Vis diffuse reflection spectrum of PCT-x-550 (x = 300, 350, 400, 450) samples is red-shifted relative to that of PCT-500-550, and a certain amount of light absorption is observed in the 400–800 nm wavelength range. This is because these samples contain a certain amount of biochar, which has a strong visible light absorption ability. This was confirmed from the UV-Vis diffuse reflection spectrum of sample PCT-0-550, which has a biochar content of approximately 69.36% and exhibits strong light absorption throughout the UV-Vis region (200–800 nm). The light absorption ability of PCT-x-550 samples decreased with the decrease in their carbon content. Furthermore, their bandgap values were estimated using the Kubelka–Munk formula. These are plotted in Figure 8b with the *x*-axis showing E = h*ν* = 1240/λ, where λ is the absorption wavelength, and (αh*ν*)^2^ on the *y*-axis [44,45,46]. As shown, the bandgap values of PCT-x-550 samples gradually increase as their bio-carbon content decreases. The approximate bandgap value of the PCT-500-550 sample was 3.20 eV, and that of the PCT-400-550 sample was 2.91 eV.

To further investigate the band structure of the samples, we conducted XPS valence band (VB) spectrum testing on sample PCT-500-550, where the carbon content could be negligible, and sample PCT-400-550, which exhibited the optimal photocatalytic activity. The valence band positions of the samples were determined using the linear extrapolation method, as illustrated in Figure 8c. It can be observed that the valence band (VB) edge of the samples PCT-500-550 and PCT-400-550 are 2.92 eV and 2.27 eV (the intersection point of the dashed line with the horizontal line), respectively. The conduction band (CB) potentials of the composite can be calculated using the empirical formula [47]: E_CB_ = E_VB_ − E _g_, where E_CB_, E_VB_, and E_g_ are the CB edge potentials, VB edge potentials, and bandgap energy, respectively. The E_CB_ potentials of samples PCT-500-550 and PCT-400-550C were calculated to be −0.28 eV and −0.69 eV, respectively. The band structure diagram of the sample was obtained from the E_VB_ and E_CB_ data, as shown in Figure 8d. It can be observed that both the VB and CB positions of the sample PCT-400-550 are higher than those of the sample PCT-500-550. This may be attributed to the formation of a heterojunction between biochar and TiO_2_.

The fluorescence spectrum is often used to investigate the separating efficiency of photogenerated electrons and holes in photocatalytic materials. The stronger the fluorescence spectrum, the lower the separating efficiency of photogenerated electrons and holes and the lower the resultant photocatalytic activity, and vice versa [48]. Thus, fluorescence tests were conducted on the TiO_2_/P, K-PC samples by considering an excitation wavelength of 320 nm [49]. The obtained results are depicted in Figure 8c. As shown, the fluorescence emission peaks of PCT-x-550 samples appeared in the vicinity of 430 nm. These spectrums were similar, and the peak intensity gradually decreased with the increase in carbon content. This indicates that the higher the carbon content in the PCT-x-550 samples, the higher the photogenerated electron–hole separating efficiency. This can be attributed to the presence of graphitic carbon in these samples, known for its excellent electrical conductivity that enhances the migration speed of photogenerated electrons. Consequently, this leads to improved separation efficiency between photogenerated electrons and holes.

The photocatalytic performance of the material is closely related to the photocurrent intensity it achieves under light. Consequently, a transient photocurrent test was conducted on TiO_2_/P, K-PC samples. The obtained results are depicted in Figure 8d. As shown, under the same test conditions, the transient photocurrent intensity values of these samples decreased with the decrease in their biochar content. This finding is consistent with the pattern reflected by their fluorescence spectra. The transient photocurrent intensity of sample PCT-0-550 is approximately twice that of sample PCT-500-550. The fluorescence spectrum and transient photocurrent analysis outcomes of all samples indicate that the inclusion of graphitic carbon promotes the enhancement of the separation efficiency between photogenerated electrons and holes in the composites, which in turn boosts their photocatalytic efficacy.

### 2.7. The Photocatalytic Performance of TiO_2_/P, K-PC Composites

**Figure 9:**
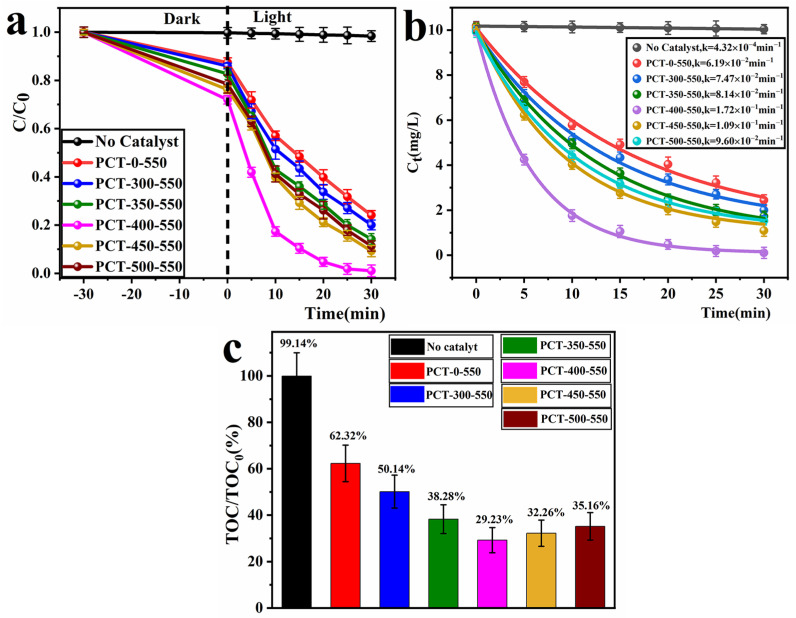
Photocatalytic degradation curves (**a**); kinetic curves (**b**) and mineralization rates (**c**) of RhB solution by PCT-x-550 samples under 300 W mercury lamp irradiation after 30 min.

To evaluate the photocatalytic performance of the fabricated TiO_2_/P, K-PC composites, RhB dye solution was used as the simulated pollutant of printing and dyeing wastewater. This experiment primarily investigated the C/C_0_~t relationship of RhB after 30 min of dark adsorption and 30 min of photocatalytic reaction, wherein the dynamic curve graphs were fitted to the first-order kinetics model according to a non-linear least square analysis, and the removal rate of total organic carbon (TOC). The experimental conditions were as follows: The light source was a 300 W mercury lamp, and the RhB concentration was 10 mg/L (100 mL). This RhB concentration was selected based on its value of 10–20 mg/L in untreated dyeing wastewater generated in factories [50]. Furthermore, the amount of photocatalyst added was 50 mg, the reaction temperature was 25 °C, and the initial pH was approximately 4.9 (unadjusted). The obtained results are depicted in Figure 9. As shown, in the reaction system without any catalyst addition, the RhB degradation rate was approximately 1.8% (Figure 9a), and the removal rate of TOC was approximately zero (Figure 9c). This indicates that the self-degradation of RhB is negligible and can be used as the simulated pollutant for photocatalytic degradation experiments. All PCT-x-550 samples showed excellent adsorption and photocatalytic performance. PCT-400-550 sample performed the best, with an adsorption rate of up to 27% after 30 min of dark adsorption and a degradation rate of 99.07% for RhB solution after 30 min of UV illumination (Figure 9a). The adsorption amounts and degradation rates of PCT-x-550 samples on RhB dye solution showed a tendency to initially increase and then decrease as their carbon content decreased. The magnitudes of these values followed the order PCT-400-550 > PCT-450-550 > PCT-500-550 > PCT-350-550 > PCT-300-550 > PCT-0-550.

Based on the photocatalytic experiments, it can be discerned that the degradation rate of the RhB solution for the PCT-x-550 (x = 0, 300, 350, 400, 450, 500) samples exhibits an increasing trend initially and then a decline with an increase in the x value. This observation suggests that the TiO_2_/P, K-PC composites demonstrate optimal photocatalytic activity only when the biochar-to-TiO_2_ mass ratio is appropriate. The incorporation of biochar helps to mitigate the recombination of photogenerated electrons and holes, thereby enhancing the material’s photoelectric efficiency and ultimately improving the photocatalytic activity of the composite. The efficiency of photocatalytic separation of electrons and holes, as well as photoelectric conversion, can be attributed to the results of the fluorescence spectrum and transient photocurrent response of the samples (Figure 8c,d). When the biochar content is high, such as in samples PCT-x-550 (x = 0, 300, 350), they demonstrate optimal performance in separating photogenerated electrons and holes, as well as in photoelectric conversion. However, the scanning electron microscope (SEM) images of samples PCT-x-550 (Figure 4) reveal that TiO_2_ nanoparticles are encapsulated by biochar. Biochar impedes TiO_2_’s absorption of light, and TiO_2_ nanoparticles block the pores of biochar, reducing both its surface area and porosity. Consequently, the contact area between the catalyst and reactants is minimized, leading to lower photocatalytic activity. When the biochar content is low, such as in samples PCT-x-550 (x = 450, 500), the efficiency of separating photogenerated electrons and holes, as well as the photoelectric conversion efficiency of the samples, is poor. Therefore, their photocatalytic activity is also low. Only when the biochar content is appropriate in TiO_2_/P, K-PC composites, such as the sample PCT-400-550, does it exhibit good efficiency in separating photogenerated electrons and holes, as well as in photoelectric conversion. TiO_2_ nanoparticles deposited on the surface of biochar enhance TiO_2_’s capacity for light absorption and conversion. Additionally, the sample PCT-400-550 possesses a larger surface area and abundant pores, which can provide more active sites for photocatalytic reactions. This increases the contact area between the catalyst and reactants, facilitating the rapid transfer of reactants and products and efficient collaboration between TiO_2_ and biochar. Therefore, sample PCT-400-550 exhibits the best photocatalytic performance.

The dynamic curve graphs were fitted to a first-order kinetics model based on a non-linear least square analysis. The formula is Ct = Xe^−kt^ + E, where Ct represents the residual concentration of calculated polluted molecules, X stands for the process’s amplitude, k denotes the pseudo-first-order rate constant, and E signifies the endpoint of the process [51]. The graphs were plotted with the x and y axes representing time t and Ct values, respectively (Figure 9b). The photodegradation rate constant k of samples PCT-x-550 (x = 0, 300, 350, 400, 450, 500) for the RhB solution were 0.0619 (R^2^ = 0.997), 0.0747 (R^2^ = 0.995), 0.0814 (R^2^ = 0.997), 0.172 (R^2^ = 0.999), 0.109 (R^2^ = 0.999), and 0.096 (R^2^ = 0.999) min^−1^. It can be observed that the k of samples PCT-400-550 was significantly higher than that of the other samples.

To further investigate the mineralization rates of the RhB dye solution by TiO_2_/P, K-PC composites. The TOC content of the RhB dye solution before the reaction and after 30 min of photocatalytic degradation was tested. According to the formula, the mineralization rate (%) = (1 − TOC/TOC_0_ ) × 100% is used to calculate the mineralization rate (Figure 9c). As shown, after 30 min of photodegradation, all the PCT-x-550 samples showed a certain removal rate of TOC from RhB dye solution, with the sample PCT-400-550 achieving the highest TOC removal rate of approximately 69%. This indicates that the molecular structure of the RhB dye solution was disrupted, and smaller molecules were generated during the photocatalytic process.

In summary, the sample PCT-400-550 outperformed the other samples in terms of photocatalytic degradation of RhB dye solution. The following investigations were primarily focused on sample PCT-400-550.

**Figure 10:**
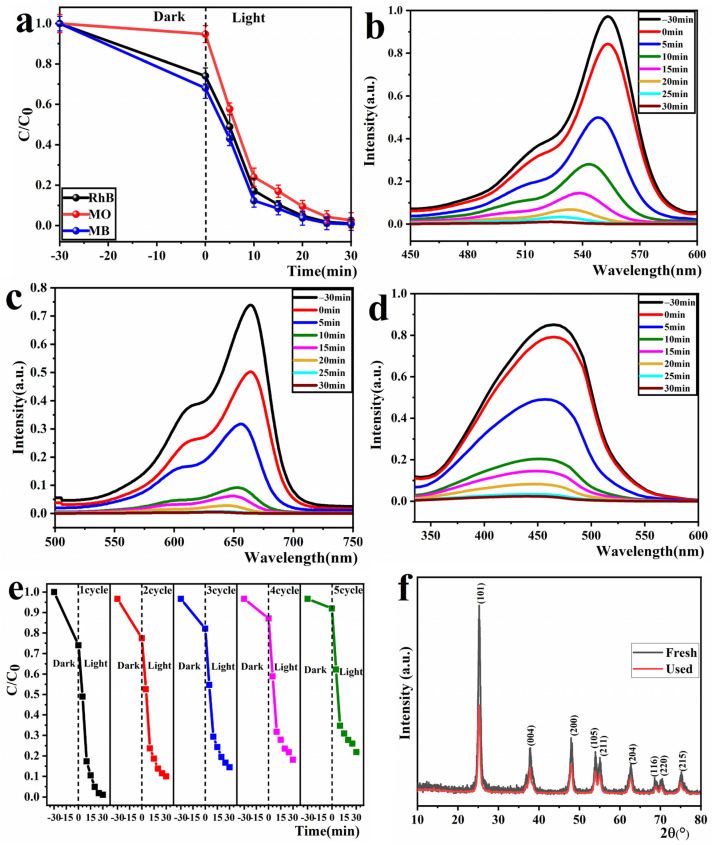
Photocatalytic degradation curves of sample PCT-400-550 for MO, RhB, and MB (**a**). Photocatalytic degradation spectra of RhB (**b**), MB (**c**), and MO (**d**) solutions. Effect of photocatalytic degradation of RhB dye solution for five cycles (**e**); XRD spectrum of the sample before and after degradation reaction (**f**).

The photocatalytic degradation experiments of 10 mg/L RhB, MO, and MB solutions were outperformed under the irradiation of a 300 W mercury lamp with the addition of 50 mg/100 mL of sample PCT-400-550. The results obtained are illustrated in Figure 10. Figure 10a demonstrates that the sample PCT-400-550 can completely photodegradation 10 mg/L RhB, MO, and MB dye solutions within 30 min. This indicates that sample PCT-400-550 has good photocatalytic applicability to dye pollutants. However, the adsorption of sample PCT-400-550 on MO dye solution was relatively small, which should be related to the electric charge of the dye chromophore group. Under neutral conditions, the chromophores of RhB and MB were positively charged, whereas the chromophore of MO was negatively charged. Thus, it can be inferred that because the PCT-400-550 sample surface was negatively charged in the case of MO, the adsorption amount of MO was much lower than that in the case of RhB and MB.

Figure 10b–d show the photocatalytic degradation spectra of RhB, MB, and MO dye solutions. As shown, as the photocatalytic reaction progressed, the intensities of the characteristic absorption peaks of RhB, MB, and MO dye solutions gradually weakened until they disappeared. This indicates that the chromophore structures in RhB, MB, and MO dye solutions were gradually destroyed during photocatalysis.

The recyclability of photocatalysts is an important index for evaluating whether they can be widely used. In this study, the PCT-400-550 sample, which showed the best photocatalytic performance, was used as an example to investigate the cyclic stability of its photocatalytic degradation of RhB dye solution. At the end of each reaction, the remaining reaction solution was centrifuged, washed, and dried to be reused for the next experiment. Fresh 10 mg/L RhB dye solution was used in each experiment, and five experiments were repeatedly performed under the same conditions. The results are depicted in Figure 10e. As shown, the adsorption and photocatalytic performance of the sample PCT-400-550 on RhB dye solution decreased after recycling five times. In particular, the adsorption performance decreased considerably, with the dark adsorption rate decreasing from 27.1% to 5.2% in 30 min. This should be because the RhB dye molecules or their degradation products blocked or destroyed the pore structure of the sample [52], which affected its adsorption performance. Furthermore, the degradation rate decreased from 99% to 81%. This can be attributed to the small catalyst loss incurred during each cycling experiment.

To further investigate the stability of the sample PCT-400-550, XRD analysis was performed on the samples before and after the cyclic reaction. The obtained results are plotted in Figure 10f. As shown, no new diffraction peaks appeared in the XRD spectrum of the sample PCT-400-550 after the cyclic reaction five times. However, the intensities of the corresponding characteristic peaks of the TiO_2_ crystal faces were weakened. This indicates that no new phases were generated in the sample PCT-400-550 after the cyclic reaction, but the active sites of TiO_2_ were passivated.

### 2.8. Photocatalytic Mechanism Research

**Figure 11:**
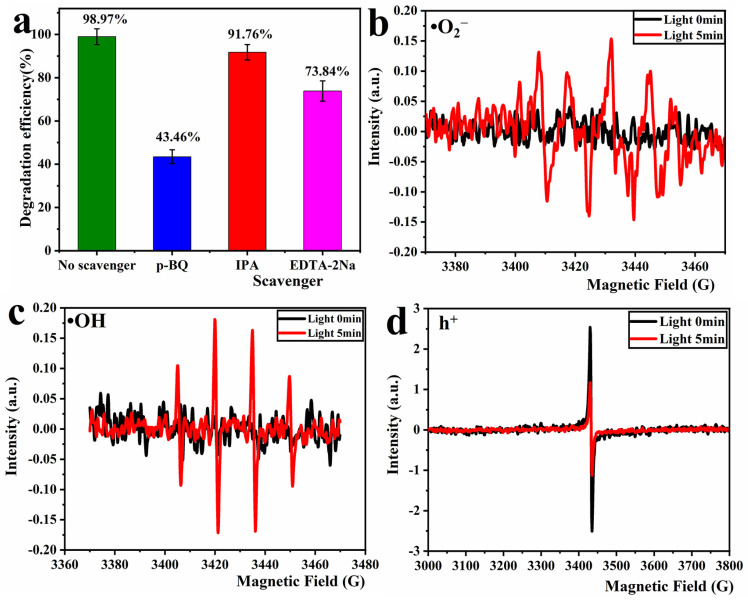
Results of free radical capture test for PCT-400-550 sample (**a**), EPR spectra for •O_2_^−^ (**b**), h^+^ (**c**), and •OH (**d**) of PCT-400-550 sample.

The mechanism of photocatalytic degradation of RhB by TiO_2_/P, K-PC composites was investigated by conducting free radical capture experiments. The lower the degradation of the dye solution after the addition of the capturing agent, the greater the role played by the captured free radical in the reaction system. In this study, we used p-benzoquinone (p-BQ) in excess to capture the superoxide anion radical (•O_2_^−^), isopropyl alcohol (IPA) to capture the hydroxyl radical (•OH), and ethylenediamine tetraacetic acid disodium salt (EDTA-2Na) to capture the hole (h^+^) [53]. The results of this experiment are depicted in Figure 11a. As shown, the RhB degradation rate was 98.97% in the comparison system without adding any capture agent, and the RhB degradation rates in the reaction systems with the addition of capture agents were all reduced to different degrees. The RhB degradation rate decreased from 98.97% to 43.46% in the reaction system with the addition of p-BQ, which was introduced to capture •O_2_^−^. This is a considerable reduction of 55.51%. In the reaction system, with the addition of IPA to capture •OH, the RhB degradation rate decreased by 7.21%. Moreover, in the reaction system, with the addition of EDTA-2Na to capture the h^+^, the RhB degradation rate decreased by 25.13%. This indicates that in the photocatalytic degradation of RhB by the sample PCT-400-550, the contribution of •O_2_^−^ is the highest, while the h^+^ and •OH also play some role.

The free radicals generated during the reaction were further verified using the EPR test, which utilized DMPO to capture •OH, •O_2_^−^, and TEMPO to verify the presence of holes. The results are depicted in Figure 11b–d. As shown, no characteristic peaks of •O_2_^−^, •OH are present in the EPR spectrum under dark conditions. After 5 min of illumination, Figure 11b shows a clear six-peak signal, which typically corresponds to DMPO-•O_2_^−^ in the methanol phase [54]. Figure 11c shows a four-peak signal with a peak intensity ratio of 1:2:2:1, which typically corresponds to DMPO-•OH in the aqueous phase [54]. Moreover, Figure 11d shows the signal peak of the capture agent TEMPO. The signal intensity after 5 min of illumination is weaker than that in the dark, indicating that the composites produce h^+^ under light. In other words, the signal intensity of TEMPO is stronger because the composites cannot produce h^+^ in darkness. During 5 min of illumination, the composites were excited by light to produce h^+^, and the reaction between h^+^ and TEMPO consumed TEMPO, which weakened the intensity of the TEMPO signal peak [55]. In summary, the sample PCT-400-550 can produce •O_2_^−^, •OH, and h^+^ under UV irradiation. All of these reactive radicals, to varying extents, contribute to the photocatalytic degradation of RhB. These three active free radicals exhibited their effects in the following order: •O_2_^−^ > h^+^ >•OH.

**Scheme 2:**
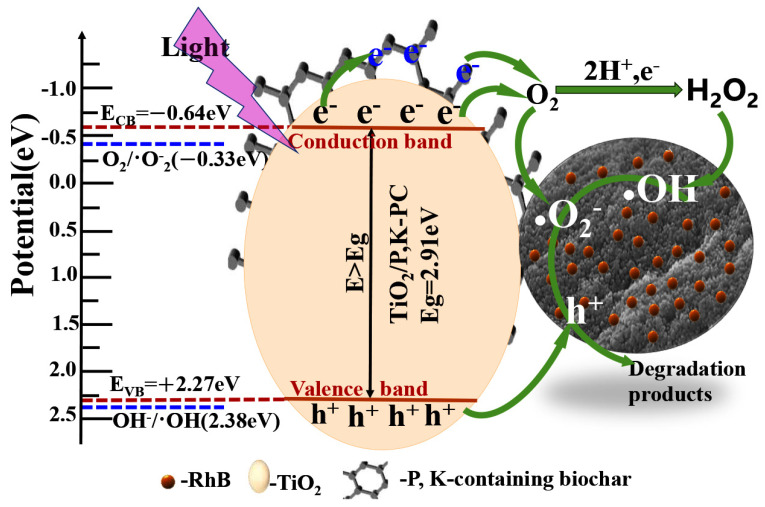
The mechanism of photocatalytic degradation of RhB by TiO_2_/P, K-PC composites.

The results of material characterization and performance analysis indicate that the improved photocatalytic performance of TiO_2_/P, K-PC composite is primarily due to their porous structure, large surface area, lower electron–hole recombination rate, the existence of P, K-containing biochar, and the synergistic effect of biochar and TiO_2_. The possible reaction mechanism is depicted in Scheme 2. When the multiphase system of catalyst and dye solution was irradiated with ultraviolet light larger than the bandgap energy, the electrons in the valence band of TiO_2_ were excited and jumped to the conduction band. Simultaneously, corresponding holes are generated in the valence band [56], forming electron–hole pairs. Moreover, because biochar has good electron transfer ability, the electrons generated by TiO_2_ were quickly transferred to biochar. This efficient charge transfer effectively improves the separation efficiency of electrons and holes. Since the conduction band (CB) potential of TiO_2_/P, K-PC composite is −0.64 eV vs. NHE, which is lower than that of O_2_/•O_2_^−^ (−0.33 eV vs. NHE), electrons will react with O_2_ adsorbed on the surface of biochar and TiO_2_ particles to form a strong oxidizing reactive free radical •O_2_^−^. Additionally, since the valence band (VB)potential of the TiO_2_/P, K-PC composite is 2.27 eV vs. NHE, lower than that of OH^−^/•OH (+2.38 eV vs. NHE), it can be inferred that h+ in the valence band will not react with OH^−^ or H_2_O to form •OH. Instead, it will directly degrade RhB owing to its potent oxidation capability [57]. However, EPR spectroscopy and free radical trapping experiments have already confirmed the presence of •OH. According to previous reports, the formation of •OH could be attributed to the reaction between O_2_ molecules and electrons, generating H_2_O_2_, which then decomposes to form •OH [58]. In the presence of •O_2_^−^, h^+^, and •OH reactive radicals, RhB molecules adsorbed on the composite material are degraded into small molecules.

The specific degradation reaction process can be described by Chemical Equations (1)–(6):

TiO_2_/P, K-PC+ hν→h^+^ + e^−^ (1)

e^−^ (TiO_2_) → e^−^ (BC) (2)

O_2_ + e^−^ (TiO_2,_ BC) → • O_2_^−^ (3)

O_2_ + 2H^+^+2e^−^ → H_2_O_2_ (4)

H_2_O_2_ + e^−^ → OH^−^ + •OH (5)

(O_2_^−^, h^+^ and •OH) + RhB → Degradation products (6)

## 3. Materials and Methods

### 3.1. Materials

Grapefruit peels were purchased from Shantou Agricultural and Trade Market, Shantou, China. The following chemicals were utilized: tetrabutyl titanate (TBOT) (500 mL, AR), rhodamine B (RhB) (100 g, AR), methylene blue (MB) (100 g, AR), and methyl orange (MO) (100 g, AR) acquired from Shanghai Macklin Biochemical Co., Ltd., Shanghai, China; as well as sodium hydroxide (500 g, AR), anhydrous ethanol (500 mL, AR) and acetic acid (500 mL, AR), both procured from Sinopharm Chemical Reagent Co., Ltd., Shanghai, China, and mercury lamp (CME-M300, China Microenergy (Beijing) Technology Co., Ltd., Beijing, China).

### 3.2. Preparation of TiO_2_/P, K-PC Composites

The yellow outer skin of grapefruit peel was peeled off. The spongy inner skin was fragmented into small pieces of approximately 1 cm and fully immersed in 0.1 mol/L NaOH solution to eliminate the cellulose within and expand the size of the cavities.

Then, these pieces were rinsed with distilled water to achieve neutrality. Next, they were dried in a blast drying oven at 60 °C for 6h, after which they were crushed and passed through a 60-mesh sieve to obtain grapefruit peel powder. Then, absolute ethanol (80 mL) was added to tetrabutyl titanate (10 mL) in a round-bottomed flask, and the pH of the solution was adjusted to pH = 2 using acetic acid. Subsequently, grapefruit peel powder (2 g) was added to the obtained mixture, and the round-bottomed flask was mounted on a modified rotary evaporator, which was rotated at 180 r/min for 12 h. The obtained mixture was then washed with absolute ethanol until its pH was neutralized, after which it was dried. Then, the grapefruit peel powder loaded with precursors was placed in a tube furnace in an air atmosphere, and the temperature was increased at a rate of 2 °C/min to certain temperatures (300 °C, 350 °C, 400 °C, 450 °C, 500 °C, respectively). At each of these stages, constant temperature was maintained for 3 h to regulate the mass ratio of TiO_2_ to carbon. Subsequently, nitrogen was introduced into the tube furnace. The temperature was increased at 2 °C/min until it reached 550 °C, which was maintained for 3 h. At this stage, the TiO_2_ was sufficiently crystallized, and biochar was sufficiently carbonized. The series of TiO_2_/P, K-containing grapefruit peel biochar composites produced using this approach were labeled in the format PCT-x-550, where x denotes the temperature of calcination of the samples under an air atmosphere, and 550 is the temperature of calcination in degrees Celsius of the samples under the N_2_ atmosphere. The samples thus obtained were labeled PCT-0-550, PCT-300-550, PCT-350-550, PCT -400-550, PCT-450-550, and PCT-500-550.

### 3.3. Photocatalytic Performance Test

The photocatalytic performance was tested using the organic dyes as organic pollutant models. These dyes included rhodamine B (RhB), methylene blue (MB), and methyl orange (MO). First, 10 mg/L (100 mL) of the dye solution was measured in the reactor, and 50 mg of the prepared sample was added to it. Then, the magnetic stirrer was turned on in the dark for 30 min first to disperse the mixture and then make it adsorbed. Subsequently, the light source and the circulating cooling water device were turned on to perform the photocatalytic degradation reaction. The light source was placed 15 cm away from the reaction solution and samples were collected every 5 min. The samples were then passed through a 0.45 μm microporous filter membrane, and their absorbance was measured to determine the change in RhB concentration until the end of the reaction. Then, the relationship of C/C_0_~t was investigated to calculate the RhB removal rate according to the Formula below.

η = (1 − C/C_0_) × 100%

where η denotes the percentage of RhB removal rate, C_0_ represents the initial RhB concentration in mol/L, and C denotes the pollutant concentration in mol/L after a certain reaction time, t.

### 3.4. Sample Characterization

The synthesized materials were subjected to a comprehensive range of techniques to assess their properties. The calcination process parameters, as well as the content of TiO_2_ and biochar in the sample, were determined using thermogravimetric analysis (TGA). The crystal structure was analyzed using X-ray diffraction (XRD), while the structure of the products was characterized using a laser Raman spectrometer with an excitation wavelength of 532 nm. Elemental composition and valence states on the surface of the material were assessed using X-ray photoelectron spectroscopy (XPS). The micro surface morphology, structure, and elemental distribution were determined using scanning electron microscopy (SEM) and energy dispersive spectroscopy (EDS). The microstructure morphology, high-resolution lattice fringes, and electron diffraction were characterized using transmission electron microscopy (TEM). Fully automated specific surface area analyzer method, BET, was used to analyze the pore size of the materials, and the light absorption properties of the samples were assessed via ultraviolet–visible diffuse reflectance spectroscopy (UV-vis DRS). The transient photocurrents of the samples were tested using an electrochemical workstation, and the photoluminescence properties were characterized using a fluorescence spectrometer. Finally, Fourier-transform infrared spectroscopy (FTIR) was employed to investigate the structural characteristics and generation mechanism of the samples. The total organic carbon present in the pollutants before and after the reaction was measured using an organic carbon analyzer to calculate the mineralization rate.

## 4. Conclusions

This study focuses on the synthesis of TiO_2_/P, K-containing grapefruit peel biochar composites using grapefruit peel as a bio-template and carbon source and tetrabutyl titanate as the titanium source. The findings indicate that the grapefruit peel played a significant role in the formation of the porous architecture of the TiO_2_/P, K-PC composites. Additionally, the content of biochar in the TiO_2_/P, K-PC composites could be adjusted by altering the calcination temperature of the sample in air. This study revealed that the sample PCT-400-550 exhibited superior photocatalytic activity under ultraviolet light irradiation towards RhB, MB, and MO. The outstanding photocatalytic performance of the sample was attributed to its unique morphological structures, abundant pores, larger surface area, and efficient collaboration between TiO_2_ and biochar, which resulted in exceptional photocatalytic performance. In conclusion, this study proposes a simple, economical, and eco-friendly strategy for the preparation of TiO_2_-biochar composites, which serves as a reference and support for the preparation of composite functional materials using biomass.

## Figures and Tables

**Table 1:** Surface area, pore volume, and average pore size of TiO_2_/P, K-PC composites.

Materials	Surface Area (m^2^·g^−1^)	Pore Volume (cm^3^·g^−1^)	Average Pore Diameter (nm)
PCT-0-550	69.40	0.11	11.9
PCT-300-550	81.27	0.17	12.0
PCT-350-550	86.73	0.17	12.2
PCT-400-550	103.84	0.24	15.4
PCT-450-550	99.80	0.22	14.5
PCT-500-550	95.79	0.19	13.4

## Data Availability

The data presented in this study are available in the Supplementary Materials.

## Supplementary Materials

The following supporting information can be downloaded at https://www.mdpi.com/article/10.3390/molecules29092090/s1, Section S1: Instrument and equipment; Scheme S1: Diagram of the preparation procedure of the TiO_2_/ P, K-PC composites.
