# Supplementary material for: Waste Biomass-Mediated Synthesis of TiO2/P, K-Containing Grapefruit Peel Biochar Composites with Enhanced Photocatalytic Activity
Source: Molecules. 2024 May 1;29(9):2090. doi: 10.3390/molecules29092090 (PMC11085262; doi:10.3390/molecules29092090)
Supplement: Supplementary file 1 [file molecules-29-02090-s001.zip › molecules-2959849-supplementary.pdf]

Supplementary Materials

Waste biomass-mediated synthesis of TiO_2/P, K-containing grapefruit peel biochar Composites with enhanced photocatalytic activity

Ruixiang Wu[1, 2],Wenhua Liu[1,*], Renao Bai[2],Delun Zheng[2],Xiufang Tian[2], Weikai Lin[2], Qianwei Ke[2], Lejian Li[2]

[1] Guangdong Provincial Laboratory of Marine Biotechnology, Institute of Marine Sciences, Shantou University, Shantou 515063, Guangdong, PR China；rxwu1@stu.edu.cn;whliu@stu.edu.cn.
2 College of Construction and Ecology, Shantou Polytechnic, Shantou, Guangdong 515078, PR China；rxwu@stpt.edu.cn；bairenao@ 163.com；xftian@ stpt.edu.cn; linweikai@163.com; qianweike1999@163.com；lilejian2024@163.com.
* Correspondence: whliu@stu.edu.cn

Corresponding authors: Wenhua Liu[1]

mail address: whliu@stu.edu.cn

Contents

SectionS1. Instrument and equipment
Scheme S1. Diagram of the preparation procedure of the TiO_2/PC composites

Section S1. Instrument and equipment

Thermal gravimetric analyzer(TG209F3,NETZSCH Scientific Instruments Trading Ltd); The waring blender (JJ-2; Changzhou Jintan Jingda Instrument Manufacturing Co., Ltd) was utilized in this research. Electronicbalance (LC-JA11003), freeze drier (LC-10N), magnetic stirrer hotplate (LC-MSA-H), desk centrifuge (TGL-LX185C) were acquired from Shanghai LiChen Instrument Technology Co., Vacuum tube furnace(GSL-1700X), provided by Hefei Kejing Materials Technology Co., Ltd， Ltd. Rotary evaporator (RE-52; Yarong Biochemical Instrument Co., Ltd); UV-Vis spectrophotometer (UV-2500 model; Shimadzu, Japan); UV diffuse reflectance spectrophotometer (UV-3600i Plus; Shimadzu, Japan); X-ray diffractometer (Ultima VI model; Rigaku Corporation, Japan); Field emission scanning electron microscope (SEM; Gemini SEM450; Carl Zeiss AG, Germany); Field emission transmission electron microscope (TEM; JEM-F200; JEOL, Japan); X-ray photoelectron spectrometer (Thermo Scientific K-Alpha; Thermo Fisher Scientific); Brunauer-Emmett-Teller (BET) surface area analyzer (ASAP 2460; Micromeritics, USA); and mercury lamp (CME-M300, China Microenergy (Beijing) Technology Co., Ltd); Fourier-transform infrared spectroscopy (FTIR, Nicolet iS50, USA); TOC analysis instrument(TOC-L,Shimadzu, Japan); Electron paramagnetic resonance(Bruker EMXnano, Bruker Corporation),Electrochemical workstation (CH1604E) provided by Shanghai Chenhua Instrument Co., Ltd., Raman spectrometer (renishaw inVa) by Renishaw plc, United Kingdom.

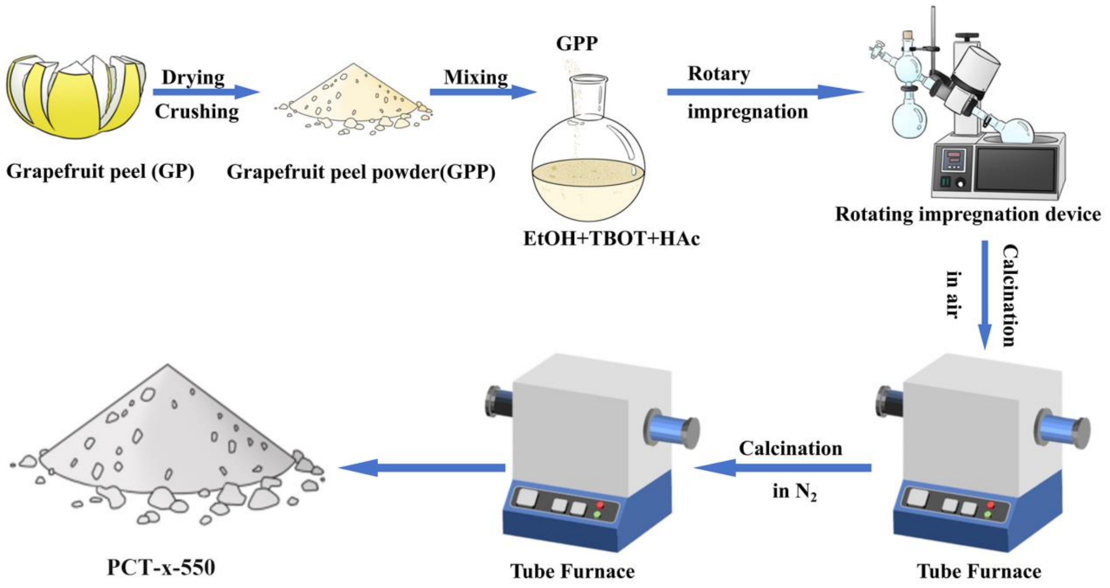

Scheme S1. Diagram of the preparation procedure of theTiO_2/PC composites
